# Free-Rider Games for Cooperative Spectrum Sensing and Access in CIoT Networks

**DOI:** 10.3390/s23135828

**Published:** 2023-06-22

**Authors:** Kejian Jiang, Chi Ma, Ruiquan Lin, Jun Wang, Weibing Jiang, Haifeng Hou

**Affiliations:** 1 Guangdong Planning and Designing Institute of Telecommunications Co., Ltd., Guangzhou 510630, China; jkj@gpdi.com; 2 College of Electrical Engineering and Automation, Fuzhou University, Fuzhou 350108, China; 200120077@fzu.edu.cn (C.M.); rqlin@fzu.edu.cn (R.L.); wangjunfzu@fzu.edu.cn (J.W.); 210110008@fzu.edu.cn (W.J.)

**Keywords:** cognitive IoT, cooperative spectrum sensing, dynamic spectrum access, game theory, energy harvesting

## Abstract

With the rapid development of technologies such as wireless communications and the Internet of Things (IoT), the proliferation of IoT devices will intensify the competition for spectrum resources. The introduction of cognitive radio technology in IoT can minimize the shortage of spectrum resources. However, the open environment of cognitive IoT may involve free-riding problems. Due to the selfishness of the participants, there are usually a large number of free-riders in the system who opportunistically gain more rewards by stealing the spectrum sensing results from other participants and accessing the spectrum without spectrum sensing. However, this behavior seriously affects the fault tolerance of the system and the motivation of the participants, resulting in degrading the system’s performance. Based on the energy-harvesting cognitive IoT model, this paper considers the free-riding problem of Secondary Users (SUs). Since free-riders can harvest more energy in spectrum sensing time slots, the application of energy harvesting technology will exacerbate the free-riding behavior of selfish SUs in Cooperative Spectrum Sensing (CSS). In order to prevent the low detection performance of the system due to the free-riding behavior of too many SUs, a penalty mechanism is established to stimulate SUs to sense the spectrum normally during the sensing process. In the system model with multiple primary users (PUs) and multiple SUs, each SU considers whether to free-ride and which PU’s spectrum to sense and access in order to maximize its own interests. To address this issue, a two-layer game-based cooperative spectrum sensing and access method is proposed to improve spectrum utilization. Simulation results show that compared with traditional methods, the average throughput of the proposed TL-CSAG algorithm increased by 26.3% and the proposed method makes the SUs allocation more fair.

## 1. Introduction

In recent years, there has been widespread interest in the automation revolution in the industrial, infrastructure, and healthcare fields through the implementation of the Internet of Things (IoT) [1]. However, available wireless spectrum resources are extremely limited, and the large-scale deployment of IoT devices will greatly exacerbate the shortage of spectrum resources and the problem of interference between devices. A large amount of data shows that the utilization of spectrum in the communication field is not high, and many authorized spectrums are not fully utilized, resulting in a huge waste of spectrum resources [2]. To address these challenges, Cognitive Radio (CR) provides an effective solution. In a CR system, Secondary Users (SUs) can access the authorized spectrum of Primary Users (PUs) with little impact on the PUs. Applying CR technology to IoT networks can effectively improve their spectrum utilization efficiency. Therefore, the design and optimization of Cognitive IoT (CIoT) networks based on CR have received considerable attention [3].

Spectrum sensing is a key technology in CR to address the problem of insufficient spectrum resources by reusing underutilized spectrum. However, the detection performance of spectrum sensing may deteriorate significantly due to uncertainties such as noise and channel conditions [4]. Cooperative Spectrum Sensing (CSS) can improve the sensing performance of fading and shadowed channels by combining the results of multiple SUs’ global sensing [5]. Specifically, due to factors such as obstruction, SUs may be located in the shadow of PU transmission signals and unable to detect their signals, which results in interference with PU’s communication. CSS can effectively address the hidden terminal problem by allowing multiple SUs to cooperate and greatly reducing the impact of shadowed areas on sensing. Furthermore, dynamic spectrum access can greatly improve the spectrum utilization efficiency between SUs and PUs [6]. Dynamic spectrum access technology allows SUs to effectively utilize spectrum resources by “leasing” or “sharing” idle spectrum resources, improving spectrum utilization, and also having significant advantages in fairness and efficiency. This technology can be used either in the overlay mode, where the SUs opportunistically utilize the unused spectrum of the PUs, or in the underlay mode, where both PUs and SUs transmit simultaneously under SINR constraints in the same frequency band [7].

Due to the limited battery capacity of IoT devices, it is difficult for them to run for a long time. Energy Harvesting (EH) allows devices to harvest energy from the environment to refill batteries [8]. For any type of energy source, the amount of energy transferred from the energy source to the energy harvester is not deterministic but random. Collecting energy through Radio Frequency (RF) signals can avoid the influence of harsh environments and light, and compared with solar energy and wind energy, the energy harvesting process is more stable. Therefore, RF EH technology has become an important direction for researchers to study the energy efficiency of CIoT [9]. However, since selfish SUs can harvest more energy for system consumption by free-riding during spectrum sensing slots, the application of EH will exacerbate the free-riding behavior of selfish SUs, leading to degraded system detection performance. Therefore, the application of EH technology needs to consider how to deal with the free-riding problem to ensure more stable and reliable system performance.

In recent years, people have been more committed to developing distributed intelligent collaboration systems due to reasons of scalability and high performance [10]. These systems consist of distributed processing nodes with autonomous learning capabilities and usually include a large number of free-riding nodes [11]. However, these free-riding nodes will result in fewer nodes providing resources, and the entire system will face the problem of insufficient utilization of resources, and the purpose of sharing will gradually disappear. In CSS, SUs report their sensing results to the FC by control channel and the final channel state is determined by the FC. However, SUs may be selfish, and they may adopt a free-riding strategy when multiple SUs occupy different sub-bands of a PU’s spectrum and can occasionally overhear the sensing results of other SUs. Free-riders tend to wait for other SUs to sense them first and take advantage of other SUs’ results so that they can reserve more time for their own data transmission. Therefore, due to the existence of free-riding behavior, there are two types of SUs with different sensing attitudes in the system: Contributing Users (CUs) who participate in cooperative sensing and Free-riding Users (FUs) who are dishonest. CUs increase the amount of information, but regardless of how much they contribute, the added information is shared equally among participants. However, if the cost of contribution exceeds the marginal benefit, SUs will be tempted not to contribute. Free-riders severely reduce the fault tolerance and content availability of the peer-to-peer system. In cooperative systems, the key to solving this problem is to establish incentive mechanisms that aim to improve utility by incentivizing nodes to become more cooperative. Ref. [12] proposed a game-theoretic framework for participating in cooperation and free-riding in federated learning to support spectrum sensing for NextG communications.

## 2. Relate Work

Spectrum sensing is the basis and key to achieving spectrum sharing, improving the efficiency of spectrum utilization, and ensuring the security of spectrum resources in CR technology. So far, many papers have applied spectrum sensing technology in IoT field. For the issue of energy efficiency in spectrum sensing, Ref. [13] investigated how to use spectrum sensing technology to improve the energy efficiency of CIoT networks, and proposed a new two-way information exchange algorithm and optimal transmit power allocation technique to achieve higher dynamic spectrum sensing capability and data throughput. In addition, cooperative spectrum sensing has been studied extensively. Ref. [14] proposes an integrated CSS and access control model to maximize the throughput of the cognitive industrial IoT by jointly optimizing the sensing time, the number of sensing nodes, and the transmit power of each node. Dynamic spectrum access is an important research direction in CIoT networks. It provides an effective way for existing radio networks to optimize system performance, enhance resource utilization, and improve system reliability. In ref. [15], the benefits of cooperative communication and cognitive radio networks (CRN) are merged to meet IoT networks’ needs. In this work, the hybrid overlay-underlay CRN is employed to guarantee both SU stability and an acceptable total throughput. In ref. [16], spectrum aware Ad hoc on-demand distance vector routing protocol is proposed without fixed base stations, aiming to address the routing needs in future IoT environments.

There are now numerous papers focusing on free-riding behavior. In ref. [17], Adar et al. analyzed the user traffic in the Gnutella system and found that there were a large number of free-riding users in the system, with only about 28% of the users sharing files. To address the problem of the behavior of free-riders in the system, Ref. [18] proposes a peer-to-peer protocol with adaptive and self-organizing topology to punish malicious peers and free-riders by considering the trustworthiness. In ref. [19], machine learning based malicious signal detection is employed for cognitive radio networks. In [20], To enhance cooperation among agents, one of the main goals of the multi-agent system is to solve the possible free-riding problem. To address this problem, the authors propose a novel strategy that allows agents to manipulate the utility of each adversary simultaneously, further promoting mutual cooperation among all agents. Although the above literature has emphasized the users’ free-riding problem, it does not consider the scheduling problem of dynamic spectrum access techniques for SUs to further improve the system’s utility.

Game theory [21] is an effective tool for analyzing optimal behavior among rational decision makers. Game theory has been widely used in distributed CR networks to effectively improve system performance and to help SUs make better use of spectrum resources. With the increasingly complex interaction behavior of IoT devices [22], single-layer networks can no longer meet the needs of the system, and the study of multilayer networks has become a key to the development of many fields. To explore the cooperative behavior among rational individuals, researchers have combined complex networks and game theory [23], which is more helpful to study the interaction behavior problem among users in complex networks. Ref. [24] explored an important tradeoff of CSS among SUs in cognitive radio networks and proposed a distributed coalition formation game based on genetic algorithms to select the optimal coalition leader to motivate SUs to actively participate. Ref. [25] proposed a potential game-based spectrum access and power control method to solve the problem of how to achieve optimal throughput in a multibeam cognitive satellite communication network. Furthermore, game theory is also widely applied to solving the free-rider problem. In ref. [26], the free-riding problem was first modeled as a public goods game. In ref. [27], the authors considered an evolutionary game model that allows SUs to choose between free-riding and normal sensing strategies.

Although [12,26,27] considered two sensing attitudes of SUs, they did not address the scheduling problem of SUs when sensing multiple PU channels, which did not work well in multi-channel cooperative spectrum sensing. In the case where SUs cooperatively sense multiple PU channels, most SUs may tend to sense the same PU channel due to geographical location and other factors, resulting in a serious shortage of SUs sensing other PU channels, which greatly affects the detection performance. This may lead to a waste of spectrum resources and even affect the normal communication of PUs. Therefore, it is necessary to consider using dynamic spectrum access to solve the scheduling problem of SUs in this case.

Different from past research, this paper focuses on the problem of SUs’ free-riding behavior in Energy Harvesting CIoT (EH-CIoT) networks. A punishment mechanism is established to stimulate SUs to sense the spectrum normally during the sensing process. For the system model with multiple PUs and SUs, a two-layered game-based cooperative spectrum sensing and access method is proposed to dynamically coordinate the strategies of SUs. Simulation results show that the proposed method significantly improves the overall throughput of the system, and the allocation of SUs in the coalition is more fair compared with traditional methods.

The rest of this paper is organized as follows: Section 3 describes the system model and time slot structure for cooperative spectrum sensing and access and gives the optimization problem modeling of this paper. Section 4 proposes a two-layer game to solve the proposed problems in Section 3. In Section 5, simulation results are presented. Finally, the conclusion is made at last in Section 6.

## 3. System Model and Problem Description

### 3.1. System Models

**Figure 1:**
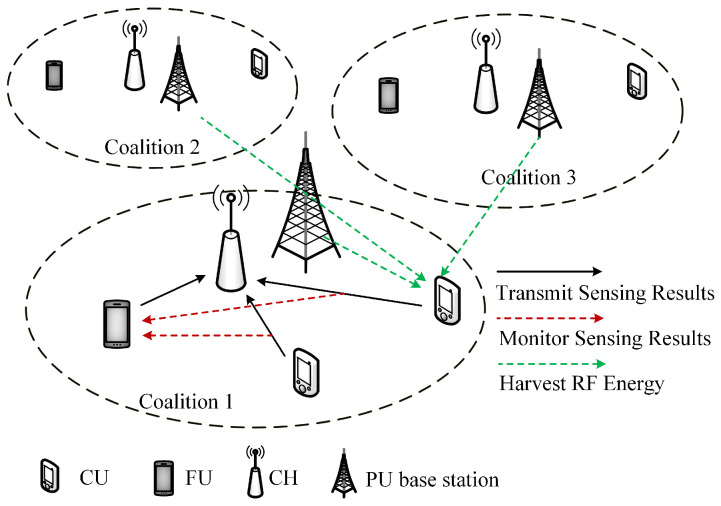
System model.

Consider an EH-CIoT network covered by *M* PU bands, which share channels with *N* SUs in overlay mode. When the PU bands are not occupied, each SU can only sense and access one band in the same time slot due to the hardware limitation. As shown in Figure 1, when SUs upload their sensing results, there is a possibility that the sensing results will be listened to by other users, and the listeners will decide to become CUs or FUs according to their utility. In each time slot, PU bands are sensed simultaneously. The SUs are divided into *M* coalitions for sensing different PU bands, and each SU can freely choose the sensing attitudes (to become CU or FU) and the band of which PU to access. In each coalition, the SU with the highest detection probability is considered the Coalition Head (CH). CH plays the role of FC in the coalition.

Due to its simplicity and low cost, the energy detector is chosen in this paper as the spectrum sensing technique to sense the PU. The noise is assumed to be an independent, identically distributed random process with a zero mean and a variance $\sigma^{2}$. In the Rayleigh fading environment, the detection probability and false alarm probability of the *i*-th secondary user ${\mathrm{SU}}_{i}$ detecting the state of the *j*-th primary user ${\mathrm{PU}}_{j}$ channel are:

(1) $$\begin{array}{cc} P_{d,i,j} & =e^{-\frac{\theta_{j}}{2}}\sum_{n=0}^{m-2}\frac{1}{n!}{\left(\frac{\theta_{j}}{2}\right)}^{n}+{\left(\frac{1+{\bar{\gamma }}_{i,j}}{{\bar{\gamma }}_{i,j}}\right)}^{m-1}\\ & \times \left[e^{-\frac{\theta_{j}}{2\left(1+\gamma_{i,j}\right)}}-e^{-\frac{\theta_{j}}{2}}\sum_{n=0}^{m-2}\frac{1}{n!}{\left(\frac{\theta_{j}{\bar{\gamma }}_{i,j}}{2\left(1+{\bar{\gamma }}_{i,j}\right)}\right)}^{n}\right] \end{array}$$

and

(2) $$\begin{array}{c} P_{f,i,j}=\frac{\Gamma \left(m,\frac{\theta_{j}}{2}\right)}{\Gamma (m)} \end{array}$$

where $\theta_{j}$ is the detection threshold of PU${}_{j}$, *m* is the time-bandwidth product, and ${\bar{\gamma }}_{i,j}$ denotes the average signal-to-noise ratio of the received signal from ${\mathrm{PU}}_{j}$ to ${\mathrm{SU}}_{i}$, Γ(.,.) is the incomplete gamma function, and Γ(.) is the gamma function. When detecting the PU channel, the probability of missing detection is:

(3) $$\begin{array}{c} P_{m,i,j}=1-P_{d,i,j} \end{array}$$

In the coalition Ω, since the control channel transmits sensing results of 0 and 1, the corresponding error reporting probability [28] can be expressed as follows:

(4) $$\begin{array}{c} P_{e,i,k}=\frac{1}{2}\left(1-\sqrt{\frac{{\bar{\gamma }}_{i,k}}{1+{\bar{\gamma }}_{i,k}}}\right) \end{array}$$

where ${\bar{\gamma }}_{i,k}$ is the average SNR for bit reporting between ${\mathrm{SU}}_{i}$ and CH${}_{k}$. The decision fusion criterion at CHs is the OR verdict criterion. By cooperative sensing, the detection probability and the false alarm probability of the coalition Ω are respectively given as follows:

(5) $$\begin{array}{c} P_{d,e}^{or}=1-\prod_{i\in \Omega }\left[P_{m,i}\left(1-P_{e,i,k}\right)+\left(1-P_{m,i}\right)P_{e,i,k}\right] \end{array}$$

and

(6) $$\begin{array}{c} P_{f,e}^{or}=1-\prod_{i\in \Omega }\left[\left(1-P_{f}\right)\left(1-P_{e,i,k}\right)+P_{f}P_{e,i,k}\right] \end{array}$$

where $P_{f}$, $P_{m,i}$ and $P_{e,i,k}$ are given by Equations (2)–(4).

### 3.2. Time Slot Structure

**Figure 2:**
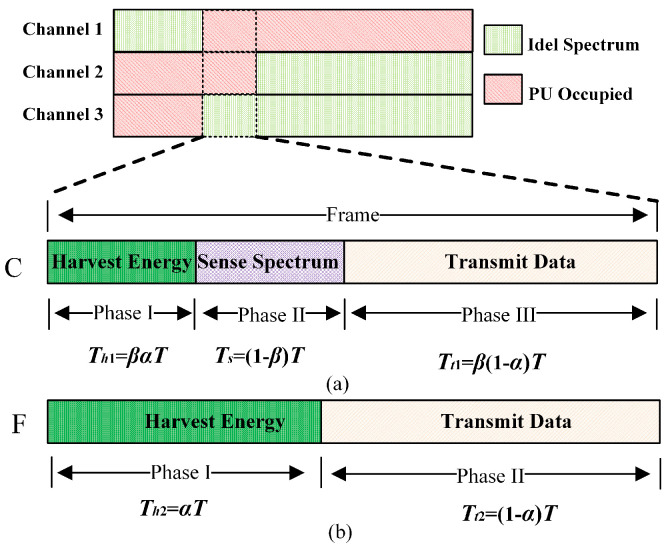
Time slot structure of the proposed method.

In this paper, the harvested energy is derived from the RF energy transmitted by the PU. As shown in Figure 2, the sensing result of the sensing phase directly affects the action of the SU in the next phase. If the PU is present, the SU harvests energy in the data transmission phase, and if the PU is not present, the SU transmits data in the data transmission phase.

CUs follow the time frame structure shown in Figure 2a, which can be divided into three phases: energy harvesting phase, the spectrum sensing phase, and the data transmission phase. FUs follow the time frame structure shown in Figure 2b and can be divided into two phases, energy harvesting phase and the data transmission phase. Compared with CUs, FUs are not involved in spectrum sensing, so they have more time to harvest energy and transmit data.

If the PU is present, according to the time slot structure, the energy harverted by CUs and FUs can be calculated separately as follows:

(7) $$\begin{array}{cc} E_{hc}^{i}\left(C\right) & =\sum_{j=1}^{M}\left(1-P_{H_{1}}^{j}\right)Q_{i}^{j}\left(T_{h1}+T_{t1}\right)\\ \; & =\sum_{j=1}^{M}\left(1-P_{H_{1}}^{j}\right)Q_{i}^{j}\left(\beta T\right) \end{array}$$

and

(8) $$\begin{array}{cc} E_{hc}^{i}\left(F\right) & =\sum_{j=1}^{M}\left(1-P_{H_{1}}^{j}\right)Q_{i}^{j}\left(T_{h2}+T_{t2}\right)\\ \; & =\sum_{j=1}^{M}\left(1-P_{H_{1}}^{j}\right)Q_{i}^{j}T \end{array}$$

If the PU is absent, according to the time slot structure, the energy harvested by CUs and FUs can be calculated separately as follows:

(9) $$\begin{array}{cc} E_{hc}^{i}\left(C\right) & =\sum_{j=1}^{M}P_{H_{1}}^{j}Q_{i}^{j}T_{h1}\\ \; & =\sum_{j=1}^{M}P_{H_{1}}^{j}Q_{i}^{j}\left(\beta \alpha T\right) \end{array}$$

and

(10) $$\begin{array}{cc} E_{hc}^{i}\left(C\right) & =\sum_{j=1}^{M}P_{H_{1}}^{j}Q_{i}^{j}T_{h1}\\ \; & =\sum_{j=1}^{M}P_{H_{1}}^{j}Q_{i}^{j}\left(\beta \alpha T\right) \end{array}$$

where $P_{H_{1}}^{j}$ is the probility of the presence of PU${}_{j}$, α and β are the weights, and 0<α,β<1, *T* is the duration of a time slot, $Q_{i}^{j}$ is the energy arrival rate at SU${}_{i}$, given by:

(11) $$\begin{array}{c} Q_{i}^{j}=P_{p_{j}}h_{j,i} \end{array}$$

where $P_{p_{j}}$ is the transmission power of PU${}_{j}$ and $h_{j,i}$ is the path loss of PU${}_{j}$ and SU${}_{i}$.

When PU${}_{j}$ is absent, and not detected by SU${}_{i}$, the average throughput of SU${}_{i}$ is

(12) $$\begin{array}{c} R_{H_{0}}=T_{t}\left(1-P_{f}\right)\log_{2}\left(1+\frac{P_{s_{i}}h_{j,i}}{\sigma^{2}}\right) \end{array}$$

When PU${}_{j}$ is present, and not detected by SU${}_{i}$, the average throughput of the SU${}_{i}$ is

(13) $$\begin{array}{c} R_{H_{1}}=T_{t}\left(1-P_{d}\right)\log_{2}\left(1+\frac{P_{s_{i}}h_{j,i}}{\sum_{j}^{M}P_{p_{j}}g_{0}+\sigma^{2}}\right) \end{array}$$

Let $P_{H_{0}}$ denotes the probability that the PU is absent, then the total throughput of SU${}_{i}$ is

(14) $$\begin{array}{cc} R_{i}= & P_{H_{0}}R_{H_{0}}^{j}+\left(1-P_{H_{0}}^{j}\right)R_{H_{1}}\\ = & T_{t}\left[P_{H_{0}}^{j}\left(1-P_{f}\right)\log_{2}\left(1+\frac{P_{s_{i}}h_{j,i}}{\sigma_{i}^{2}}\right)\right.\\ \; & \left.+\left(1-P_{H_{0}}^{j}\right)\left(1-P_{d}\right)\log_{2}\left(1+\frac{P_{s_{i}}h_{j,i}}{\sum_{j}^{M}P_{p_{j}}g_{0}+\sigma_{i}^{2}}\right)\right] \end{array}$$

In dynamic spectrum access, it is required that the SUs’operation should not conflict or interfere with the PUs, and $P_{d}$ should be very close to one. Moreover, we usually have $\sum_{j}^{M}P_{p_{j}}g_{0}+\sigma_{i}^{2}>\sigma_{i}^{2}$ due to the interference from the PUs to the SUs, the second term on the RHS of (14) is much smaller than the first term on the RHS of (14). To simplify the formulation, the total throughput of SU${}_{i}$ access PU${}_{j}$ channel can be expressed as follows:

(15) $$\begin{array}{cc} R_{i} & =P_{H_{0}}^{j}R_{H_{0}}\\ \; & =T_{t}P_{H_{0}}^{j}\left(1-P_{f}\right)\log_{2}\left(1+\frac{P_{s_{i}}h_{j,i}}{\sigma_{i}^{2}}\right) \end{array}$$

where $P_{H_{0}}^{j}$ denotes the probability that the PU${}_{j}$ is not present and $R_{H_{0}}$ denotes the average throughput of SU${}_{i}$ when PU${}_{j}$ is not present. $T_{t}$ is the duration of the data transmission phase in a time slot. According to Equation (15), the throughput $R_{i}^{C}$ of CU${}_{i}$ can be expressed as follows:

(16) $$\begin{array}{c} R_{i}^{C}=T_{t1}P_{H_{0}}^{j}\left(1-P_{f}\right)\log_{2}\left(1+\frac{P_{s_{i}}h_{j,i}}{\sigma_{i}^{2}}\right) \end{array}$$

where $T_{t1}=\beta \left(1-\alpha \right)T$, $P_{s_{i}}$ is the transmission power of SU${}_{i}$. Similarly, the throughput of FU${}_{i}$ can be expressed as follows:

(17) $$\begin{array}{c} R_{i}^{F}=T_{t2}P_{H_{0}}^{j}\left(1-P_{f}\right)\log_{2}\left(1+\frac{P_{s_{i}}h_{j,i}}{\sigma_{i}^{2}}\right) \end{array}$$

where $T_{t2}=\left(1-\alpha \right)T$.

### 3.3. Optimization Problem Modeling

The SUs aim to accomplish a joint task, which is to protect the PU from interference, achieve a required detection threshold for fusion detection probability through CSS, and obtain high throughput by accessing the PU spectrum. CUs can eavesdrop on sensing results and have more time for their own data transmission. However, if a sufficient number of SUs do not sense the PU channel, all of them may get very low throughput. Therefore, the SUs need to try different strategies in each time period and learn the optimal strategy from their strategic interactions.

The utility of SUs consists of four components: the benefit obtained based on throughput, the benefit obtained from harvesting energy, the cost of consuming energy during data transmission, and the penalty for free-riders based on the coalition detection probability. Considering that FUs do not contribute to the coalition detection probability, a penalty needs to be imposed on them. The penalty function can be expressed as follows:

(18) $$\begin{array}{c} \chi \left(P_{d}^{j}\right)=\lambda \min\left\{1,-\log S\left(P_{d}^{j}\right)\right\} \end{array}$$

where λ is a predetermined parameter defining the harshness of the penalty, and $P_{d}^{j}$ is the detection probability of detecting PU${}_{j}$. The sigmoid function for the satisfaction degree of the detection performance is calculated as follows:

(19) $$\begin{array}{c} S\left(P_{d}^{j}\right)=\frac{1}{1+e^{-\rho \left(P_{d}^{j}-{\tilde{P}}_{d}\right)}} \end{array}$$

where ${\tilde{P}}_{d}$ is the predefined requirement for the uncertainty, and ρ decides on the steepness of the satisfactory curve. The utility functions of CUs and FUs can be defined as follows:

(20) $$\begin{array}{c} U_{i}\left(C\right)=f_{c}\left(R_{i}^{C}\right)-E_{t1}+E_{hc}^{i}\left(C\right) \end{array}$$

and

(21) $$\begin{array}{c} U_{i}\left(F\right)=f_{c}\left(R_{i}^{F}\right)-E_{t2}-\chi \left(P_{d}^{j}\right)+E_{hc}^{i}\left(\;F\right) \end{array}$$

where the first term $f_{c}\left(x\right)$ on the RHS of Equations (20) and (21) is the satisfaction function of each SU with respect to the achievable throughput, and for simplicity we choose to set $f_{c}\left(x\right)=\mu x$, where μ is the uniform unit parameter for the SUs to convert the throughput into the corresponding benefit, $E_{t1}$ and $E_{t2}$ are the energy consumed by CUs and FUs during data transmission. Then Equations (20) and (21) can be expressed as follows:

(22) $$\begin{array}{cc} U_{i}\left(C\right) & =\mu \beta \left(1-\alpha \right)TP_{H_{0}}^{j}\left(1-P_{f}\right)\log_{2}\left(1+\frac{P_{s_{i}}h_{j,i}}{\sigma_{i}^{2}}\right)\\ \; & -P_{H_{0}}^{j}P_{s_{i}}\beta \left(1-\alpha \right)T\\ \; & +\sum_{j=1}^{M}\left[P_{H_{0}}^{j}Q_{i}^{j}\left(\beta T\right)+\left(1-P_{H_{0}}^{j}\right)Q_{i}^{j}\left(\beta \alpha T\right)\right] \end{array}$$

and

(23) $$\begin{array}{cc} U_{i}\left(F\right) & =\mu \left(1-\alpha \right)TP_{H_{0}}^{j}\left(1-P_{f}\right)\log_{2}\left(1+\frac{P_{s_{i}}h_{j,i}}{\sigma_{i}^{2}}\right)\\ \; & -P_{H_{0}}^{j}P_{s_{i}}\left(1-\alpha \right)T-\lambda \min\left\{1,-\log S\left(P_{d,i}\right)\right\}\\ \; & +\sum_{j=1}^{M}Q_{i}^{j}\left[P_{H_{0}}^{j}T+\left(1-P_{H_{0}}^{j}\right)\left(\alpha T\right)\right] \end{array}$$

To improve the utility of SUs, it is crucial to increase the system throughput and enhance the cooperative detection probability to reduce penalties, as indicated Equations (20) and (21), and it can be inferred that system throughput is primarily influenced by the false alarm probability. Increasing the false alarm probability can improve the system’s throughput. In order to improve SUs’ throughput, the problem of whether SUs participate in cooperative sensing and which coalitions SUs choose to access is formulated as an optimization problem. Equations (22) and (23) need to be optimized simultaneously, and the problem formulation is given as follows:

(24a)max{Pdj},{Pfj}Ui(C),Ui(F)∀i=1,2,…,N(24b)s.t.0≤β≤1(24c)0≤α≤1

## 4. Two-Layer Cooperative Sensing and Access Game (TL-CSAG)

**Figure 3:**
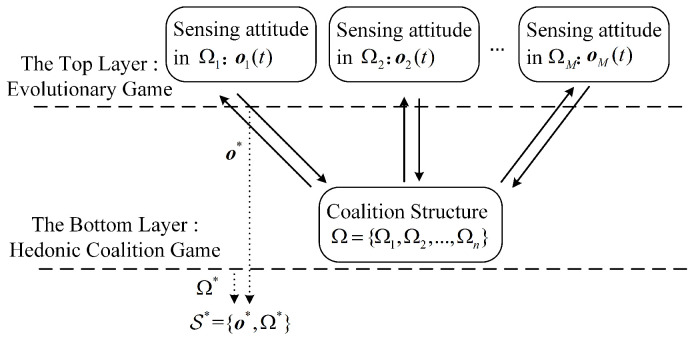
The structure of TL-CSAG model.

As shown in Figure 3, based on evolutionary game and hedonic coalition game models, an iterative algorithm for solving the game problem mentioned above is proposed in this section. In this paper, the cooperative spectrum sensing and access mechanism is modeled as a two-layer game, where the problem of whether SUs participate in cooperative sensing is modeled as an evolutionary game and the problem of which coalitions SUs choose to access is modeled as a hedonic coalition game. In this game, the SUs are considered participants, and $U_{i}$ is considered a utility function of SU${}_{i}$. The SUs’ sensing attitude preference in the coalitions can be described as follows:

(25) $$\begin{array}{c} o\left(t\right)=\left[\begin{array}{c} o_{1}\left(t\right)\\ o_{2}\left(t\right)\\ \vdots \\ o_{N}\left(t\right) \end{array}\right]=\left[\begin{array}{cccc} o_{1,1}\left(t\right) & o_{1,2}\left(t\right) & \ldots & o_{1,M}\left(t\right)\\ o_{2,1}\left(t\right) & o_{2,2}\left(t\right) & \ldots & o_{2,M}\left(t\right)\\ \vdots & \vdots & \ddots & \vdots \\ o_{N,1}\left(t\right) & o_{N,2}\left(t\right) & \ldots & o_{N,M}\left(t\right) \end{array}\right] \end{array}$$

where $o_{i,j}\left(t\right)$ denotes the sensing attitude preference of SU${}_{i}$ in coalition $\Omega_{j}$ in the *t*-th time slot, $o_{i,j}\left(t\right)\in \left\{0,1\right\}$. $o_{i,j}\left(t\right)=1$ indicates that SU${}_{i}$ will choose to sense PU${}_{j}$ channel in coalition $\Omega_{j}$, and $o_{i,j}\left(t\right)=0$ indicates that SU${}_{i}$ will choose to be a free-rider in coalition $\Omega_{j}$. The SU${}_{i}$’s strategy is denoted by $s_{i}=\left(o_{i},\omega_{i,j}\right)$ and $\omega_{i,j}$ denotes the SU${}_{i}$ choose the coalition $\Omega_{j}$. To provide a clearer depiction of how SUs can modify their utility through specific actions, its utility function can be rewritten as follows:

(26) $$\begin{array}{c} U_{i}\left(s_{i}\right)=U_{i}\left(o_{i},\omega_{i,j}\right)=\left\{\begin{array}{cc} U_{i}\left(C\right), & \;\mathrm{if}\;o_{i,j}=1\\ U_{i}\left(F\right), & \;\mathrm{if}\;o_{i,j}=0 \end{array}\right. \end{array}$$

Each SU maximize its utility by optimizing its sensing attitude and then enter a coalition. Thus, the optimization problem is formulated as follows:

(27) $$\begin{array}{c} \begin{array}{cc} \max_{\left\{o_{i}\right\},\left\{\omega_{i,j}\right\}} & U_{i}\left(o_{i},\omega_{i,j}\right)\;\forall i=1,\ldots ,N;\forall j=1,\ldots ,M\\ \;s.t.\; & \left\{\begin{array}{c} 0⩽\beta ⩽1\\ 0⩽\alpha ⩽1 \end{array}\right. \end{array} \end{array}$$

From Equations (24)–(27), it can be seen that the strategies of both problems are interdependent. Therefore, the interaction of SUs can be formulated as a noncooperative game. Under the condition of Nash Equilibrium (NE), each SU’s strategy cannot get better utility without changing the strategy of other SUs. To comprehensively consider the feasibility and convergence of the two-level game algorithm, the definition of NE is given as follows:

**Definition** **1.** *Let $o^{*}=\left(o_{1}^{*},\ldots ,o_{N}^{*}\right)$ and $\omega^{*}=\left(\omega_{1}^{*},\ldots ,\omega_{N}^{*}\right)$ be the NEs of the sensing attitude strategy and the coalition formation strategy derived by the proposed TL-CSAG, respectively. $s^{*}=\left(\left(o_{1}^{*},\omega_{1}^{*}\right),\ldots ,\left(o_{N}^{*},\omega_{N}^{*}\right)\right)$, is an NE of TL-CSAG, and it can be given by:*

(28) $$\begin{array}{c} U_{i}\left(s_{i}^{*},s_{-i}^{*}\right)\geq U_{i}\left(s_{i},s_{-i}^{*}\right),\forall s_{i}^{*}\in s^{*},i\in N \end{array}$$

*where $s_{i}^{*}=\left(o_{i}^{*},\omega_{i}^{*}\right)$, $s_{-i}^{*}$ is the stable strategy for all SUs except SU${}_{i}$.*

### 4.1. The Top Layer Game: Sensing Attitude Strategy in the Coalition

This subsection analyzes the evolution of SUs’ sensing attitudes using the idea of evolutionary game theory. In each time slot, SUs calculate their own utility. If SU${}_{i}$’s utility ${\bar{U}}_{ij}$(C) for participating in sensing in $\Omega_{j}$ is higher than the average utility ${\bar{U}}_{ij}$, then in the next time slot, the probability of SU${}_{i}$ participating in sensing will increase. To describe the evolution of the sensing attitude of SU${}_{i}$ in $\Omega_{j}$, the following differential equations [29] are constructed as follows:

(29) $$\begin{array}{c} {\dot{P}}_{i}^{j}\left(C\right)=\frac{P_{i}^{j}\left(C,t+1\right)-P_{i}^{j}\left(C,t\right)}{P_{i}^{j}\left(C,t\right)}=\eta_{i}\left[{\bar{U}}_{i}^{j}\left(C\right)-{\bar{U}}_{i}^{j}\right] \end{array}$$

where $\eta_{i}$ is the adjustment step size determined by SU${}_{i}$. $P_{i}^{j}\left(C,t\right)$ is the probability that in the *t*-th time slot SU${}_{i}$ participates in sensing in $\Omega_{j}$.

In the next time slot, the probability of SU${}_{i}$ participation in sensing in $\Omega_{j}$ can be calculated as follows:

(30) $$\begin{array}{c} P_{i}^{j}\left(C,t+1\right)=P_{i}^{j}\left(C,t\right)+\eta_{i}\left[{\bar{U}}_{i}^{j}\left(C\right)-{\bar{U}}_{i}^{j}\right]P_{i}^{j}\left(C,t\right) \end{array}$$

Equation (30) describes the dynamic process of ${\mathrm{SU}}_{i}$ choosing its sensing attitude, where the sum of the probabilities of participating in sensing and free-riding is equal to 1.

This paper first study the game of two SUs in $\Omega_{j}$, i.e., $\Omega_{j}=\left\{g_{1},g_{2}\right\}$. The payoff table of the two SUs is shown in Table 1 according to Equations (19) and (20), where $A=1-P_{f}^{C}$, $B_{i}=1-P_{fi}$, $D_{i}=\mu \left(1-\alpha \right)TP_{H_{0}}^{j}\log_{2}\left(1+\frac{P_{s_{i}}h_{j,i}}{\sigma_{i}^{2}}\right)$, $E_{i}=\sum_{j=1}^{M}\left[P_{H_{0}}^{j}Q_{i}^{j}T+\left(1-P_{H_{0}}^{j}Q_{i}^{j}\alpha T\right)\right]$, $F=P_{H_{0}}^{j}P_{p_{j}}\left(1-\alpha T\right)$, $W_{i}=P_{H_{0}}^{j}P_{s_{i}}\left(1-\alpha T\right)+\lambda \min\left\{1,-\log S\left(P_{d,i}\right)\right\}$.

Let $x_{1}$ and $x_{2}$ denote the probability that $g_{1}$ and $g_{2}$ take attitude “C”, respectively, and $V_{i}=E_{i}-F$, then the expected payoff ${\bar{U}}_{g_{1}}\left(C\right)$ while $g_{1}$ chooses to contribute is expressed as follows:

(31) $$\begin{array}{c} {\bar{U}}_{g_{1}}\left(C\right)=\beta \left(AD_{1}+V_{1}\right)x_{2}+\beta \left(B_{1}D_{1}+V_{1}\right)\left(1-x_{2}\right) \end{array}$$

The mean utility function of $g_{1}$ is given by:

(32) $$\begin{array}{cc} {\bar{U}}_{g_{1}} & =\beta \left(AD_{1}+V_{1}\right)x_{1}x_{2}+\beta \left(B_{1}D_{1}+V_{1}\right)x_{1}\left(1-x_{2}\right)\\ \; & +\left(B_{2}D_{1}+E_{1}-W_{1}\right)\left(1-x_{1}\right)x_{2} \end{array}$$

Therefore, the replicator dynamics of the two SUs using Equation (26) will be:

(33) $$\begin{array}{c} G\left(x_{1}\right)=x_{1}\left(1-x_{1}\right)\left(G_{1}x_{2}+H_{1}\right) \end{array}$$

and

(34) $$\begin{array}{c} F\left(x_{2}\right)=x_{2}\left(1-x_{2}\right)\left(G_{2}x_{1}+H_{2}\right) \end{array}$$

where $H_{i}=\beta \left(B_{i}D_{i}+V_{i}\right)$, $G_{1}=\beta \left(AD_{1}-B_{1}D_{1}\right)-\left(B_{2}D_{1}+E_{1}-W_{1}\right)$ and $G_{2}=\beta \left(AD_{2}-B_{2}D_{2}\right)-\left(B_{1}D_{2}+E_{2}-W_{2}\right)$. According to the conditions of equilibrium, we have $\dot{x_{1}}=0$ and $\dot{x_{2}}=0$, then, we get four equilibrium points: (0,0),(0,1),(1,0),(1,1), and the mixed strategy equilibrium $\left(-\frac{E_{2}}{G_{2}},-\frac{E_{1}}{G_{1}}\right)$. The Jacobian matrix is formed by taking the partial derivatives of Equations (38) and (39), then we can obtain:

(35) $$\begin{array}{c} J_{m}=\left[\begin{array}{cc} \frac{\partial G\left(x_{1}\right)}{\partial x_{1}} & \frac{\partial G\left(x_{1}\right)}{\partial x_{2}}\\ \frac{\partial F\left(x_{2}\right)}{\partial x_{1}} & \frac{\partial F\left(x_{2}\right)}{\partial x_{2}} \end{array}\right] \end{array}$$

where

(36) $$\begin{array}{c} \left\{\begin{array}{c} \frac{\partial G\left(x_{1}\right)}{\partial x_{1}}=\left(1-2x_{1}\right)\left(G_{1}x_{2}+H_{1}\right)\\ \frac{\partial G\left(x_{1}\right)}{\partial x_{2}}=G_{1}x_{1}\left(1-x_{1}\right)\\ \frac{\partial F\left(x_{2}\right)}{\partial x_{1}}=G_{2}x_{2}\left(1-x_{2}\right)\\ \frac{\partial F\left(x_{2}\right)}{\partial x_{2}}=\left(1-2x_{2}\right)\left(G_{2}x_{1}+H_{2}\right) \end{array}\right. \end{array}$$

From the stability conditions of the Jacobian, the system converges when det$(J_{m})>0$ and tr$(J_{m})<0$. The stability conditions for the four equilibrium points are:

(1) When $\beta <\frac{D_{2}B_{1}+V_{2}-W_{2}}{D_{2}A+V_{2}-W_{2}}$, the strategies of $g_{1}$ and $g_{2}$ converge to (C, F).

(2) When $\beta <\frac{D_{1}B_{2}+V_{1}-W_{1}}{D_{1}A+V_{1}-W_{1}}$, the strategies of $g_{1}$ and $g_{2}$ converge to (F, C).

(3) When $\beta >\frac{D_{2}B_{1}+V_{2}-W_{2}}{D_{2}A+V_{2}-W_{2}}$ and $\beta >\frac{D_{1}B_{2}+V_{1}-W_{1}}{D_{1}A+V_{1}-W_{1}}$, the strategies of $g_{1}$ and $g_{2}$ converge to (C, C).

(4) When $\beta <\frac{D_{2}B_{1}+V_{2}-W_{2}}{D_{2}A+V_{2}-W_{2}}$ and $\beta <\frac{D_{1}B_{2}+V_{1}-W_{1}}{D_{1}A+V_{1}-W_{1}}$, the strategies of $g_{1}$ and $g_{2}$ converge to (C, F) or (F, C) based on the initial adopted strategies.

The above mentioned has demonstrated the $o^{*}$ of the two SUs. However, due to the different utility functions of different SUs, it is difficult to obtain $o^{*}$ in the case of multiple SUs. Through repeated games, SUs can autonomously adapt to the changing environment, and each SU has a clear sensing strategy.

### 4.2. The Bottom Layer Game: Coalition Formation Strategy

Regardless of what sensing attitude is adopted or which coalition is accessed, it will directly affect the utility of SUs. In the bottom layer game, the method used by SUs to solve the problem of which coalition to access is modeled as a hedonic coalition game. Next, the relevant definitions and related theorems are stated and proved.

**Definition** **2.** *(Switch Rule): Given a partition $\Pi =\{\Omega_{1},\ldots ,\Omega_{m},\ldots \Omega_{M}\}$ of SUs’ set N, SU${}_{i}\in \Omega_{m}$ decides to leave it current coalition $\Omega_{m}$ and join another coalition $\Omega_{m^{\prime }}\in \Pi$, where $m\neq m^{\prime }$, if and only if $\Omega_{m^{\prime }}⋃\left\{i\right\}{≻}_{i}\Omega_{m}$, where ${≻}_{i}$ is the preference relation of $SU_{i}$. As a result, $\left\{\Omega_{m},\Omega_{m^{\prime }}\right\}\to \left\{\Omega_{m}\setminus \left\{i\right\},\Omega_{m^{\prime }}⋃\left\{i\right\}\right\}$.*

In order to evaluate the preferences of SU${}_{i}$ over its own sets of possible coalitions, the concept of preference relation is introduced [30] as follows:

(37) $$\begin{array}{c} \Omega_{m^{\prime }}{⪰}_{i}\Omega_{m}\Leftrightarrow u_{i}^{\Omega_{m^{\prime }}}\geq u_{i}^{\Omega_{m}} \end{array}$$

where the relationship of $\Omega_{m^{\prime }}{⪰}_{i}\Omega_{m}$ means that SU${}_{i}$ prefers to join coalition $\Omega_{m^{\prime }}$ over coalition $\Omega_{m}$. The $u_{i}^{\Omega_{m}}$ which is the preference function of SU${}_{i}$ in $\Omega_{m}$ can be expressed as follows:

(38) $$\begin{array}{c} u_{i}^{\Omega_{m}}=\left\{\begin{array}{cc} U_{i}^{\Omega_{m}}\left(o_{i,m},\Omega_{m}\right), & \;\mathrm{if}\;\Omega_{m}\notin h\left(i\right)\\ -\infty , & \;\mathrm{otherwise}\; \end{array}\right. \end{array}$$

where $U_{i}^{\Omega_{m}}$ is the utility of $SU_{i}$ in coalition $\Omega_{m}$ and h(i) is the history set of $SU_{i}$. The history set contains the coalitions that SU${}_{i}$ has joined prior to formation of the current partition Π. In general, considering the history set can accelerate the convergence of the system.

According to the preference relationship of SU in Equation (34), it can be considered that when the history set of SU${}_{i}$ is not considered:

(39) $$\begin{array}{c} \Omega_{m^{\prime }}{≻}_{i}\Omega_{m}\Leftrightarrow U_{i}\left(o_{i,m^{\prime }},\Omega_{m^{\prime }}\right)>U_{i}\left(o_{i,m},\Omega_{m}\right) \end{array}$$

**Theorem** **1.** *In TL-CSAG, the convergence rate of the sensing attitude strategy of SUs always precedes that of the coalition formation strategy. That is, the formation of $o^{*}$ precedes the formation of $\omega^{*}$.*

**Proof.**  When the sensing attitude strategy does not reach $o^{*}$, according to Equation (26), the utility function of SUs has two states, and the utility is unstable. According to the description of Equation (39), when SU${}_{i}$ exchanges its coalition, it may have $U_{i}\left(o_{i,m^{\prime }},\Omega_{m^{\prime }}\right)>U_{i}\left(o_{i,m},\Omega_{m}\right)$ and will join coalition $\Omega_{m^{\prime }}$. However, in the subsequent time slots, due to the change of $o^{*}$, $U_{i}\left(o_{i,m^{\prime }},\Omega_{m^{\prime }}\right)>U_{i}\left(o_{i,m},\Omega_{m}\right)$ may occur, and SU${}_{i}$ will switch coalition again.    □

After o reaches $o^{*}$, SUs determines their possible strategy of obtaining high returns $o^{*}$, and will focus on the distribution of SUs in the coalition. When a division $\Pi^{*}$ is a NE, it means that there is no coalition that makes the SU strictly like to join, while the other coalitions are not hurt by the formation of this new coalition. The NE formed by the hedonistic coalition is specifically defined as follows:

**Definition** **3.** *A parition $\Omega^{*}=\left\{\Omega_{1}^{*},\Omega_{2}^{*},\ldots ,\Omega_{M}^{*}\right\}$ is NE if $\forall SU_{i}\in \Omega_{j}^{*}\;\mathrm{with}\;\forall j\in M,\Omega_{j}^{*}{⪰}_{i}\Omega_{l}⋃\left\{i\right\},\forall l\in M$.*

**Theorem** **2.** *Once the sensing attitudes of SUs reache the stability strategy $o^{*}$, the final coalition will also achieve the stability strategy $\Pi^{*}$.*

**Proof.**  After each switching operation, SUs will obtain higher utility in the new coalition. Given the number of channels *M* in the CIoT network and the number of SUs *N*, the total number of different partitions is $M^{N}$, which is a finite number. Thus, from any given initial partition $\Pi_{0}$, the switching operation always terminates at some point after a finite number of iterations, where the coalition structure converges to the final partition $\Pi^{*}$.    □

### 4.3. Algorithm Steps

Based on the analysis of the above two parts, the whole process of the proposed cooperative spectrum sensing and access algorithm based on the two-layer game can be obtained. During the initialization phase, each coalition selects the SU with the highest detection probability among all SUs as its CH. Furthermore, once a CH is selected by a coalition, it cannot be chosen by any other coalition, and it must always have a cooperative attitude. Furthermore, $o^{*}$ of each coalition’s sensing attitude strategy is obtained by the evolution among the SUs. On the basis of $o^{*}$, the $\omega^{*}$ formed by SUs can be obtained through the switch rules, and the specific steps are shown in Algorithm 1.
**Algorithm 1:** The proposed two-layer cooperative sensing and accsee game**Require:** Location of the SU and the PU**Ensure:** Stable sensing attitudes $o^{*}$; Stable sensing coalitions $\Pi^{*}$;1:Initialization: Set iterations t=1, initial sensing strategy probability $P_{0}=50\%$, initialize parameter μ and η; Randomly initialize the attitudes of SUs $o_{0}$; Randomly and evenly distribute the SUs to each coalition $\Pi_{0}$;2:**while**$s\neq s^{*}$ and t∈1:MAX **do**3: t=t+1;4: Randomly select two coalitions $\Omega_{n}$ and $\Omega_{m}$ from the coalitions set Π, and then switch SU${}_{i}\in \Omega_{n}$ to the coalition $\Omega_{m}$, etc., $\left\{\Omega_{n},\Omega_{m}\right\}\to \left\{\Omega_{n}^{\prime },\Omega_{m}^{\prime }\right\}$ = $\left\{\Omega_{n}\setminus \left\{i\right\},\Omega_{m}⋃\left\{i\right\}\right\}$;5: SU${}_{i}$ chooses a strategy $o_{i,m}$ with probability $P_{i}^{m}\left(C,t\right)$;6: Calculate the utility $U_{i}\left(o_{i,n},\Omega_{n}\right)$ of SU${}_{i}$, and calculate the utility $U_{i}\left(o_{i,n},\Omega_{m}^{{}^{\prime }}\right)$ of the coalition $\Omega_{m}^{{}^{\prime }}$ at which SU${}_{i}$ is exchanged;7: SU${}_{i}$ decides whether to join a new coalition based on Equation (37);8: Update $P_{i}^{n}\left(C,t\right)$ and $P_{i}^{m}\left(C,t\right)$ based on Equations (29) and (30);9: **while** $o=o^{*}$ **do**10:  Set t=t+1;11:  Repeat steps 4, 6, 7;12:  Set $o\leftarrow o^{*}$;13: **end while**14:**end while**

## 5. Simulation Analysis and Evaluation

In this section, MATLAB is used to simulate the performance of the proposed method. The spatial environment is established in a 1 km × 1 km planar coordinate system with 3 PUs and 15 SUs randomly distributed in the coordinate system; the sampling frequency is $f_{s}=1$ MHz, the bandwidth is 2 MHz and the time of each frame is T = 10 ms. The rest of the simulation parameters are shown in Table 2. The method proposed in [31] is used here as a comparison, which is denoted by “Con”. The random algorithm randomizes the sensing attitudes and the coalitons of all SUs, which are denoted by “Random”. In the following section, the final state distribution of SUs, the relationship between detection probability and cooperation probability, which represents the proportion of contributors out of 15 SUs, the factors affecting cooperation probability, the fairness of the SUs distribution in the coalition, and finally the performance of the three algorithms in terms of throughput are investigated.

**Figure 4:**
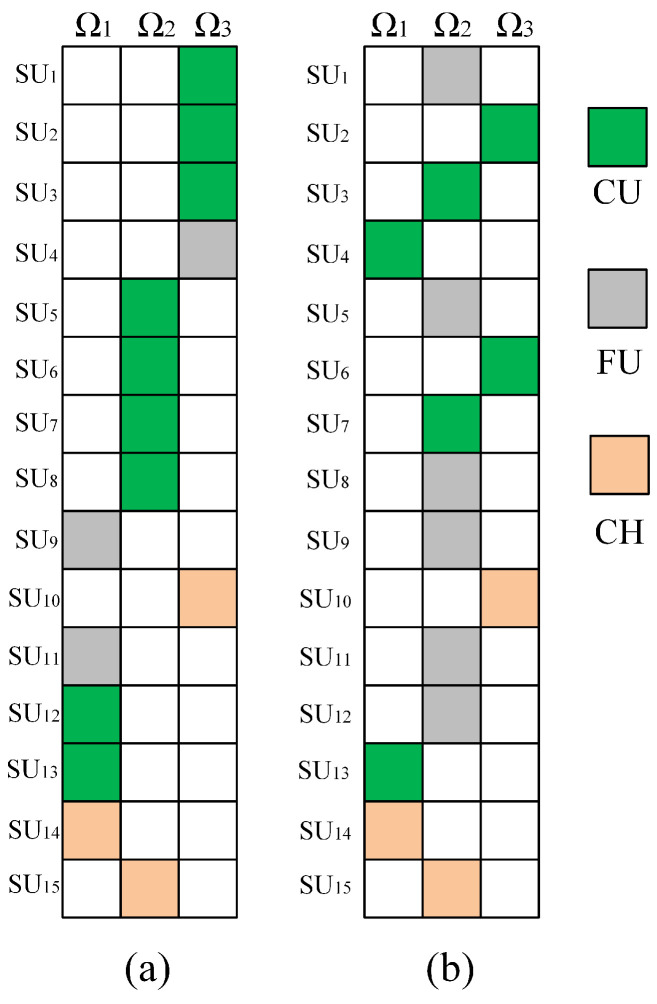
(**a**) Initial and (**b**) Final coalition structures.

The initial coalition structure and the final coalition structure obtained from TL-CSGA are shown in Figure 4a,b, respectively. In these two figures, each colored box represents an SU, and the boxes in the same column belong to the same coalition. As can be seen from Figure 4b, SUs are finally distributed in three coalitions, and FUs are all distributed in coalition $\Omega_{2}$. When the CIoT network is stable, $P_{f}$ and $P_{d}$ for each coalition will be fixed. As FUs do not contribute to the two parameters, switching their coalitions after the system is stable will not affect the interests of other SUs. Eventually, all FUs will be concentrated in the coalition with the highest benefit. Since the number of CUs will affect $P_{f}$, it is not advisable for each coalition to have too many CUs; otherwise, it will reduce the throughput of the system. Generally, CUs will be roughly evenly distributed in each coalition.

**Figure 5:**
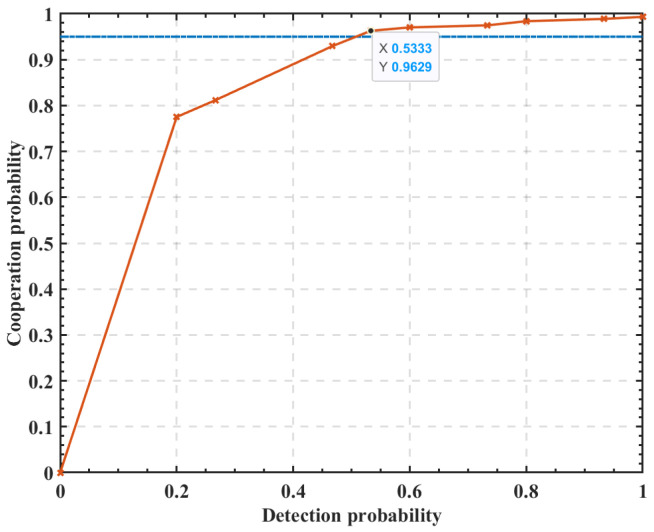
The relationship between cooperation probability and detection probability.

Figure 5 shows the relationship curves between the cooperation probability and the detection probability. As can be seen from Figure 5, with a certain number of SUs, the average detection probability of coalitions increases as the cooperation probability increases. In CSS, to avoid interference from SUs to PUs, the coalition detection probability $P_{d}$ needs to reach a certain threshold of $\theta_{th}$, i.e., $P_{d}>\theta_{th}$. As a certain number of SUs participate, when the cooperation probability reaches a certain value, the coalition detection probability $P_{d}$ can meet the requirement of $P_{d}>\theta_{th}$. This paper assumes that the coalition detection probability needs to meet $P_{d}>0.95$.

**Figure 6:**
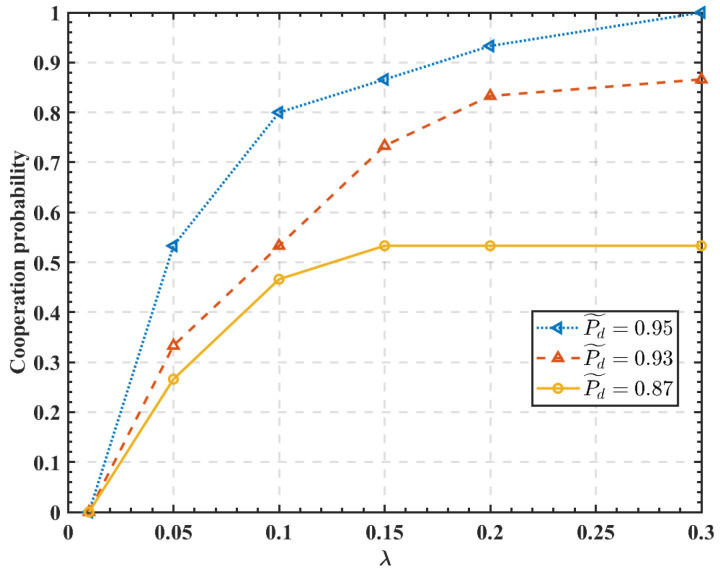
Effect of the parameters λ and ${\tilde{P}}_{d}$ on the cooperation probability.

Figure 6 shows the effect of λ and ${\tilde{P}}_{d}$ on the probability of cooperation with the 15 SUs. It can be seen that the increased value of λ is more likely to prompt SUs to adopt a cooperative sensing attitude. Moreover, it may also increase the probability of SUs cooperation with increasing ${\tilde{P}}_{d}$ values.

**Figure 7:**
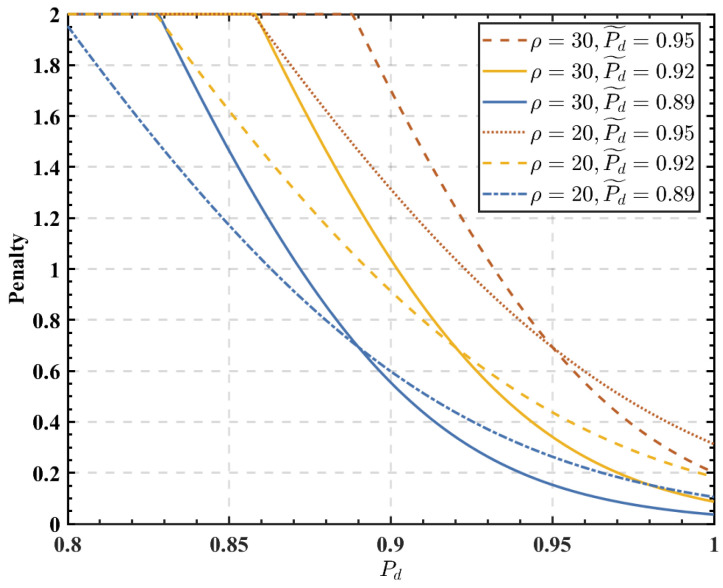
Effect of the coalition detection probability on the user penalty.

Figure 7 shows the effect of ρ, ${\tilde{P}}_{d}$, and detection probability $P_{d}$ on the penalty function. As shown in Figure 7, the higher the detection probability of the coalition, the smaller the penalty to FUs in the coalition. For the same detection probability, decreasing ${\tilde{P}}_{d}$ will reduce the penalty to FUs. In Figure 6, the effect of ${\tilde{P}}_{d}$ on the cooperation probability is essentially the effect of ${\tilde{P}}_{d}$ on the penalty function, which in turn affects the cooperation probability of SUs.

**Figure 8:**
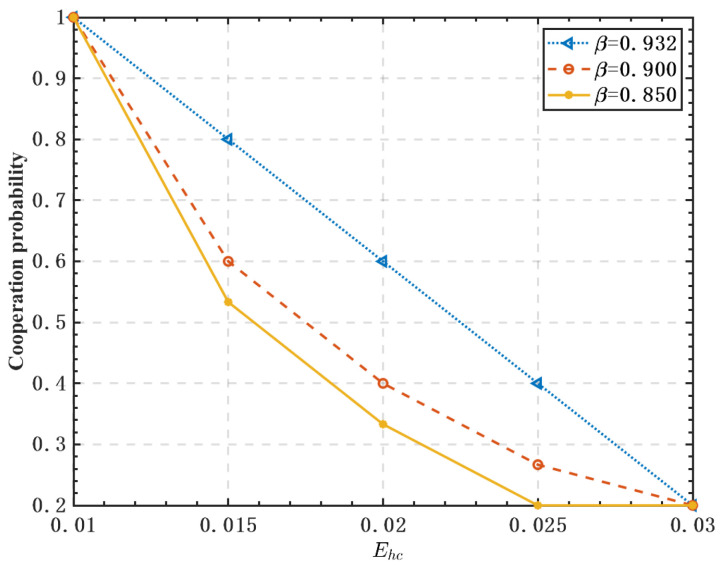
Effect of the energy harvested by FUs on the cooperation probability.

Figure 8 shows the effect of the energy harvested by FUs and the weight factor β on the cooperation probability of the system. In Figure 8, it can be observed that the more energy harvested by FUs, the lower the cooperation probability. The reason is that free-riders can obtain high profits by harvesting more RF energy. Similarly, a lower weight factor β leads to a lower probability of cooperation. Under a certain amount of energy harvesting by FUs, increasing β can improve energy harvesting benefits, reduce consumption during spectrum sensing, and improve data transmission efficiency. The simulation results show that the EH technology can provide more benefits to selfish SUs, and its application may exacerbate their free-riding behavior.

In order to evaluate the fairness of SUs’ allocation of each coalition, Jain’s fairness is introduced [32], which is defined as follows:

(40) $$\begin{array}{c} F=\frac{{\left(\sum_{i=1}^{n}x_{i}\right)}^{2}}{n\sum_{i=1}^{n}x_{i}^{2}} \end{array}$$

Jain’s fairness evaluates the fairness of a set of values, where there are *n* coalitions and $x_{i}$ is the value allocated to SU${}_{i}$, and it is located in [1/N,1]. 1/N corresponds to the minimum fair allocation where only one SU obtains a non-zero value, and 1 corresponds to the maximum fair allocation where all SUs receive the same value.

By adjusting the cooperation probability to ensure $P_{d}>0.95$, the fairness of different algorithms in SUs’ allocation was analyzed. The fairness indices of the three coalitions in Figure 4b are shown in Table 3, which lists the detection probabilities, false alarm probabilities, and corresponding fairness F of the three coalitions using TL-CASG and “Con” algorithms. It can be seen that the fairness index of the TL-CASG algorithm is superior to that of the “Con” algorithm in terms of both detection probability and false alarm probability. This is because the “Con” algorithm only considers the distance between SUs and PUs when forming coalitions without considering the influence of SUs in other coalitions globally.

**Figure 9:**
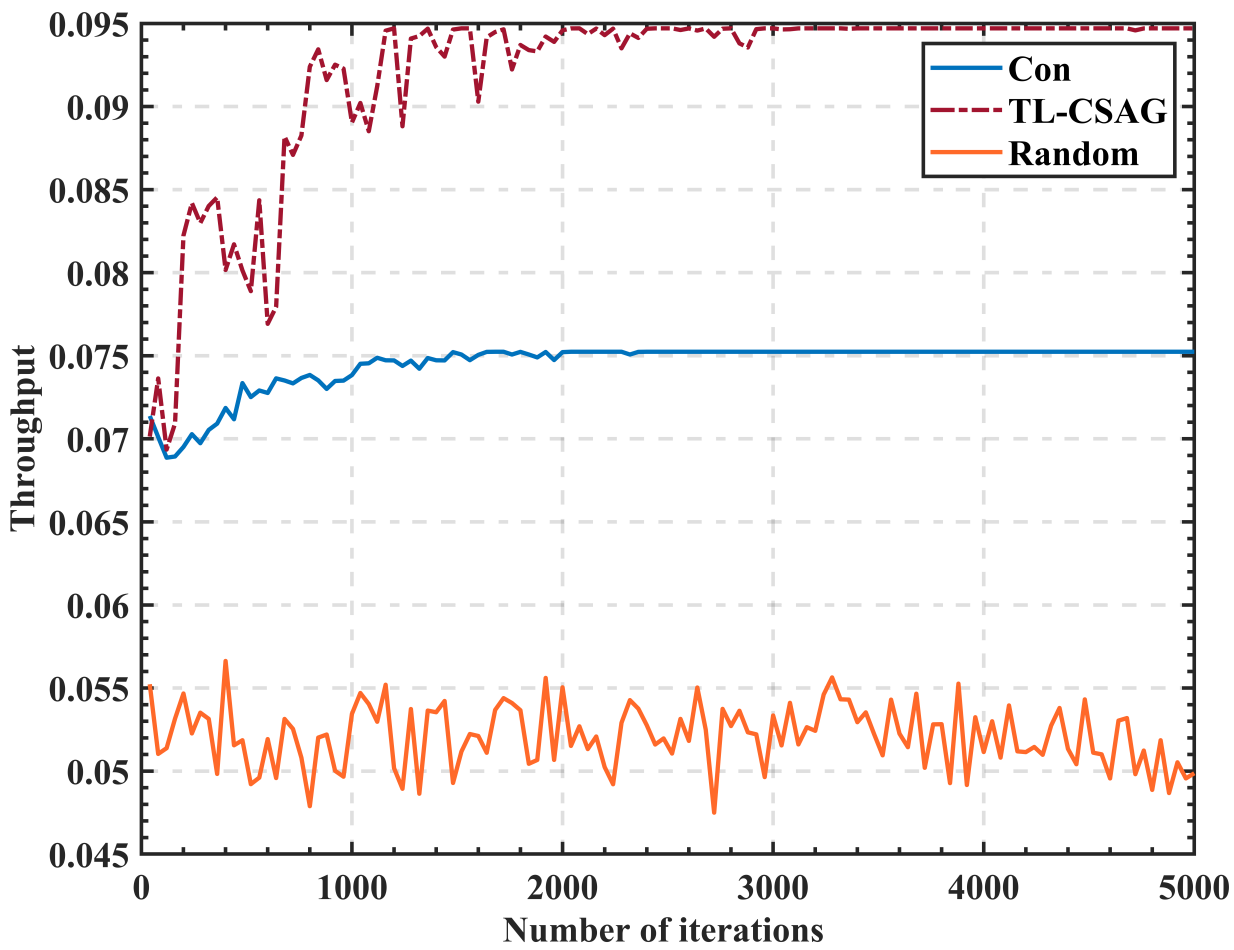
Curve of throughput with number of iterations.

The relationship between throughput and the number of iterations of three algorithms is compared in Figure 9. By modeling the problem of which PU channel is sensed by SUs, the proposed algorithm can fully utilize the sensing capability of SUs in the multi-PU model and improve the overall system throughput. After reaching an equilibrium state in the “Con” algorithm and TL-CSAG, the average throughput of the proposed TL-CSAG algorithm increased by 78% and 26.3%, respectively, compared with the “Random” algorithm and “Con” algorithm.

## 6. Conclusions

On the basis of the EH-CIoT model, this paper considers the free-riding problem of SUs. In order to prevent low system detection performance due to excessive SUs’ free-riding behavior, this paper establishes a penalty mechanism to stimulate SUs to sense the PU spectrum normally. To address the issue of whether SUs should free-ride and which PU spectrum to sense and access to gain higher benefits, a two-layer game-based cooperative spectrum sensing and access method is proposed to improve spectrum utilization and the fairness of the proposed method. Simulation results show that after the system reaches equilibrium, CUs are uniformly distributed among the three coalitions, but FUs are in the coalition with the highest profit. In the case of a fixed number of SUs, as the probability of cooperation increases, the average detection probability of coalitions also increases. At the same time, this paper also studied the impact of different parameters on the probability of cooperation, and simulation results confirmed that the more energy harvested, the lower the probability of SUs adopting a cooperative sensing attitude. Furthermore, Jain’s fairness concept is introduced to evaluate the higher fairness of the coalition in SUs’ allocation in our algorithm. Finally, this paper studied the impact of using different algorithms on throughput, and the results show that the proposed algorithm improves throughput by 78% and 26.3% compared with traditional algorithms. In the future study, we will further explore the secrecy and energy-efficient resource allocation for our proposed network. In the future study, we will further explore the application of free-riders in mobile networks and optimize algorithm models to achieve faster iteration speeds.

## Figures and Tables

**Table 1:** Payoff table of a two-user.

	*C*	*F*
C	$\beta \left(AD_{1}+E_{1}-F\right),\beta \left(AD_{2}+E_{2}-F\right)$	$\beta \left(B_{1}D_{1}+E_{1}-F\right),B_{1}D_{2}-W_{2}+E_{2}$
F	$B_{2}D_{1}-W_{1}+E_{1},\beta \left(B_{2}D_{2}+E_{2}-F\right)$	0, 0

**Table 2:** Simulation parameters.

Parameter	Meaning	Value
*m*	time bandwidth product	5
λ	the parameter to determine the value of penalty	0.02
μ	the parameter to determine the value of revenue	10
η	adjustment step size	3
$\sigma^{2}$	Gaussian noise variance	$1\times 10^{\left(-9\right)}$ W
$P_{p_{j}}$	PU transmission power	0.1 W
$P_{s_{i}}$	SU transmission power	0.1 W
$P_{H_{0}}$	probability that the PU is absent	0.9
β	weighting factor	0.9
α	weighting factor	0.5
ρ	penalty gradient coefficient	20

**Table 3:** Coalition fairness analysis.

Coalition	$P_{d-\mathrm{TL}-\mathrm{CSAG}\;}$	$P_{d-\mathrm{Con}\;}$	$P_{f-\mathrm{TL}-\mathrm{CSAG}\;}$	$P_{f-\mathrm{Con}\;}$
$\Omega_{1}$	0.9729	0.9653	0.2849	0.3751
$\Omega_{2}$	0.9846	0.9934	0.1611	0.1718
$\Omega_{3}$	0.9613	0.9583	0.2139	0.4139
FairnessF	0.9999	0.9997	0.9495	0.9002

## Data Availability

Not applicable.
